# Facile Synthesis of Calcium Borate Nanoparticles and the Annealing Effect on Their Structure and Size

**DOI:** 10.3390/ijms131114434

**Published:** 2012-11-08

**Authors:** Maryam Erfani, Elias Saion, Nayereh Soltani, Mansor Hashim, Wan Saffiey B. Wan Abdullah, Manizheh Navasery

**Affiliations:** 1 Department of Physics, Faculty of Science, University Putra Malaysia, 43400UPM Serdang, Selangor, Malaysia; E-Mails: elias@science.upm.edu.my (E.S.); nayereh.soltani@gmail.com (N.S.); mansor@science.upm.edu.my (M.H.); navaseri@gmail.com (M.N.); 2 Department of Non-ionizing Radiation, Malaysian Nuclear Agency (Nuclear Malaysia), Bangi, 43000 Kajang, Malaysia; E-Mail: wansaffiey@nuclearmalaysia.gov.my

**Keywords:** calcium borate, nanoparticles, co-precipitation, annealing time, annealing temperature

## Abstract

Calcium borate nanoparticles have been synthesized by a thermal treatment method via facile co-precipitation. Differences of annealing temperature and annealing time and their effects on crystal structure, particle size, size distribution and thermal stability of nanoparticles were investigated. The formation of calcium borate compound was characterized by X-ray diffraction (XRD) and Fourier Transform Infrared spectroscopy (FTIR), Transmission electron microscopy (TEM), and Thermogravimetry (TGA). The XRD patterns revealed that the co-precipitated samples annealed at 700 °C for 3 h annealing time formed an amorphous structure and the transformation into a crystalline structure only occurred after 5 h annealing time. It was found that the samples annealed at 900 °C are mostly metaborate (CaB_2_O_4_) nanoparticles and tetraborate (CaB_4_O_7_) nanoparticles only observed at 970 °C, which was confirmed by FTIR. The TEM images indicated that with increasing the annealing time and temperature, the average particle size increases. TGA analysis confirmed the thermal stability of the annealed samples at higher temperatures.

## 1. Introduction

In the last two decades, a burst of research activities has been devoted to nanomaterials. It has attracted many works in various fields from material science to biotechnologies and genetics [1–3]. The science and technology on such a small realm, namely nanoscience and nanotechnology, are multi-disciplinary burgeoning fields involving functional systems whose structures and components, due to their small size, hold extraordinary properties different from their bulk and molecular counterparts [4]. These novel properties are due to a large number of active atoms at their surfaces and the three-dimensional confinement of electrons [5].

The inherently high surface-to-volume ratio of nanocrystals offers the possibility of a significant contribution of the surface states to act as centers for luminescent quenching and photo bleaching. Currently, reports on luminescence properties of nanomaterials has revealed that they include some outstanding characteristics such as high sensitivity and linearity dose response over a wide range of absorbed doses [6–12] and they can be counted as a promising results for future of radiation monitoring devices in the protection area or medical and industrial applications. Therefore, significant progress has been focused on development of new materials for radiation monitoring and for this alkali and alkaline rare earth tetraborates have been found as suitable candidates in this application due to their effective atomic number, which is very close to that of human tissue (*Z*_eff_ = 7.42) and they can be used in medical application [13]. The properties of these materials are greatly influenced by the characteristics of the component, such as chemical composition, purity, particle size, and morphology [14–18], which can be controlled during the synthesis process.

In recent years, different physical and chemical methods are developed for preparation of nano phosphors materials. Among different known techniques of production, the most popular techniques are wet chemical techniques including sol gel [19], hydrothermal [20], precipitation and co-precipitation [10,21–23] and solution combustion synthesis [12,24,25] rather than conventional solid state method that usually applied to prepare bulk materials [13,26–29]. The conventional solid state reaction method leads to several shortcoming such as high sintering temperatures, poor homogeneity and larger grain size growth [18,30]. Nonetheless, the silent features of wet chemical techniques are that the starting materials can be mixed at molecular level and the temperature of formation of the final products is lower than the conventional solid-state reaction techniques. Moreover, the previous reports confirmed that the wet chemical techniques could be efficiently control the morphology and chemical composition of synthesized nanomaterial products [14,18,30]. On the other hand, among wet chemical methods, sol-gel, hydrothermal and colloidal emulsions are time consuming and involve highly unstable alkoxides and difficult to control the reaction conditions. Co-precipitation is one of successful methods for production of ultrafine nanosize ceramic powders having narrow particle size distribution [14].

This paper reports the synthesis of ultrafine calcium borate (CaB_4_O_7_) nanoparticles using co-precipitation method followed by thermal treatment method. Polyvinyl pyrrolidone (PVP) was used as the capping agent during synthesis process in order to control the size and reduce agglomeration of the particles. The synthesized nanoparticles were characterized using X-ray diffraction (XRD) and Fourier Transform Infrared spectroscopy (FTIR), Transmission electron microscopy (TEM), and Thermogravimetry (TGA).

## 2. Results and Discussion

### 2.1. XRD Analysis

**Figure 1:**
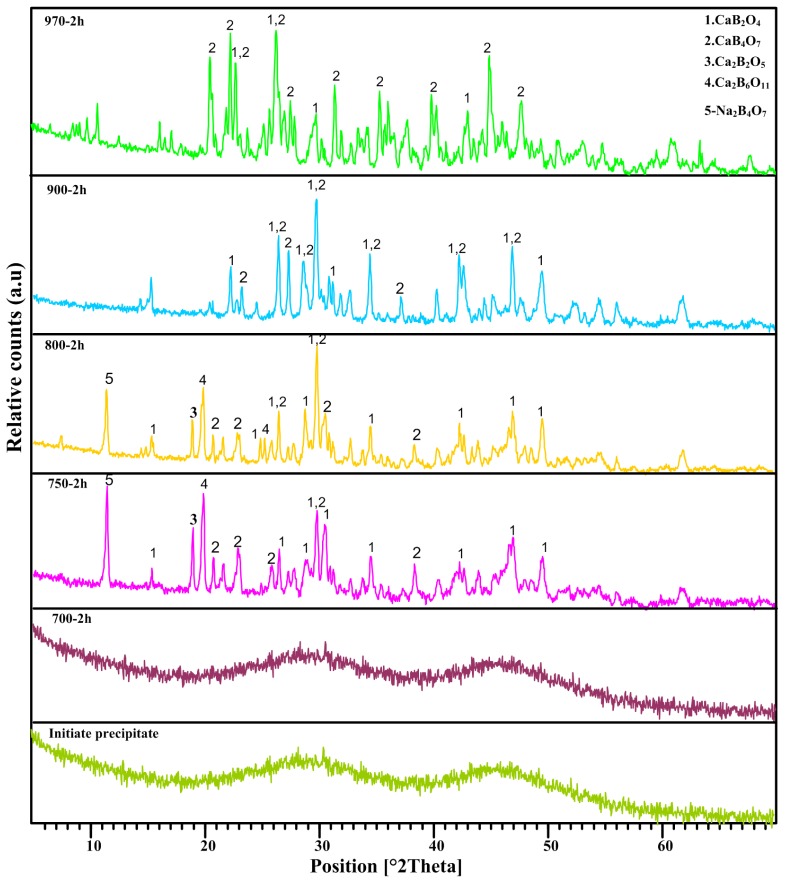
The X-ray diffraction (XRD) patterns of calcium borate annealed at 2 h for different temperatures. The amorphous structure remained at 700 °C and crystalline structure started to grow at 750 °C.

The formation of calcium borate compound was confirmed by XRD studies. Figure 1 shows the XRD patterns of samples prepared in various annealing temperatures for a fixed time of 2 h. The XRD patterns showed the amorphous structure for the initial precipitated and annealed sample at 700 °C. For higher annealing temperatures, transformation to crystalline phase started at 750 °C with appearance the dominate phase of CaB_2_O_4_ along with other phases, while, the transformation to CaB_4_O_7_ phase can be obtained at 970 °C. As can be seen, the annealing temperatures of 750 °C and 800 °C shows one peak belong to initial precursor (Na_2_B_4_O_7_) which is diminished with increasing the temperature. Furthermore, with increasing the annealing temperature from 750 °C to 900 °C, the dominate phase of CaB_2_O_4_ is still remaining and at higher temperatures, other phases of Ca_2_B_2_O_5_and Ca_2_B_6_O_11_ disappeared and some new peaks belong to metaborate (CaB_2_O_4_) and tetra borate (CaB_4_O_7_) become manifest. The main diffraction peaks belong to dominate phase at different annealing temperatures at fixed time of 2 h is presented in Table 1.

The XRD peaks presented at 2*θ* values of 26.47°, 29.75°, 32.97° and 45.18° matching with the (111), (210), (220) and (022) crystalline planes of the orthorhombic structure of calcium metaborate (ICDD PDF 32-0155) and the peaks observed at 2*θ* values of 24.42°, 26.092°, 26.47°, 29.75°, 34.65°, 38.27°, 42.25° and 46.89° are belong to calcium tetraborate (ICDD PDF 83-2025) at annealing temperature of 970 °C.

**Figure 2:**
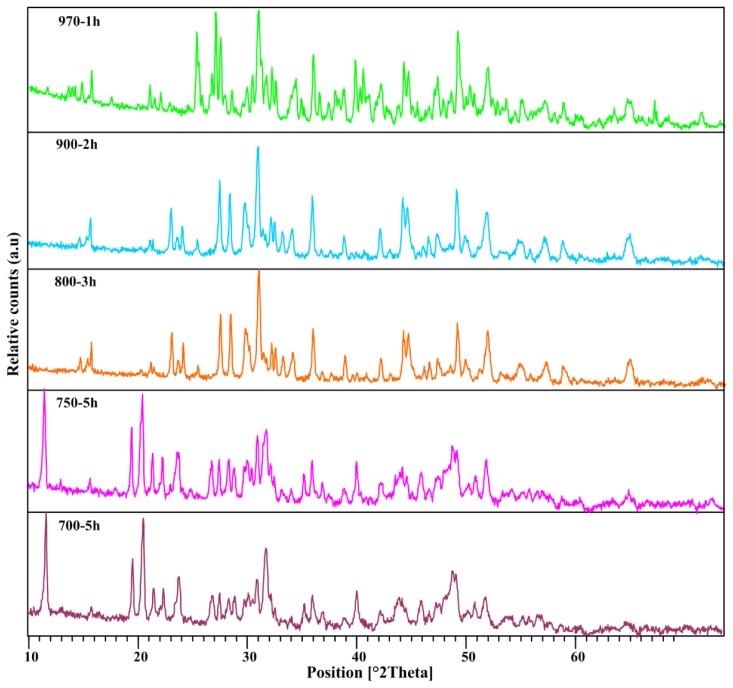
The XRD pattern structure of calcium borate for different times and temperatures.

To monitor the effect of annealing time on phase formation and properties of the samples, the heating processes were carried out in extended time up to 5 h. The XRD patterns of samples annealed at different times for a range of temperatures from 700 °C up to 970 °C are shown in Figure 2. The results showed that with increasing the annealing time at 700 °C, the conversion of amorphous to crystalline phase started at 5 h. However, the changing of annealing time for the temperatures between 750 °C–970 °C, does not show any significant changes in crystal structure (Table 2). It seems that the effect of temperature on crystal phase formation is more predominance than annealing time (Table 2). As the annealing time increases, the diffraction peaks become sharper and narrower, and the intensity increases which indicates that the intensification in crystallinity.

### 2.2. FTIR Analysis

**Figure 3:**
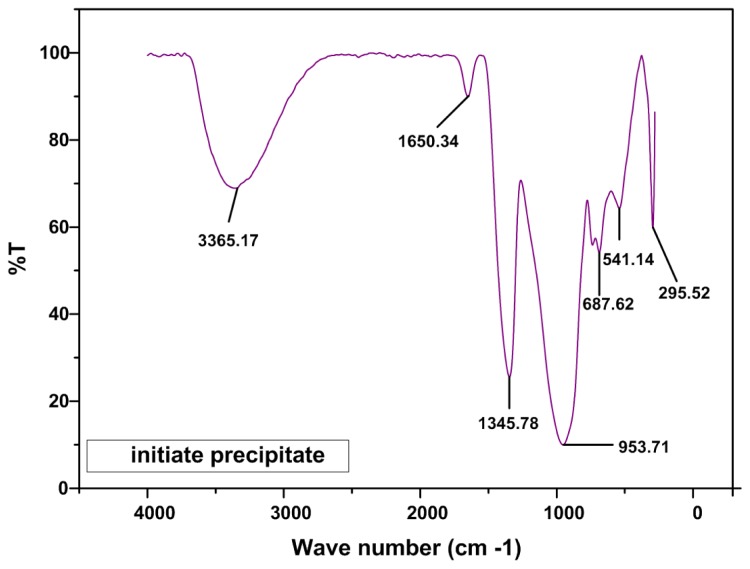
Infrared (IR) spectra of calcium tetra borate (CaB_4_O_7_) nanoparticles for initiate precipitate.

Fourier transform infrared spectroscopy (FT-IR) was employed as an auxiliary characterization alternative. The spectra of calcium borate were obtained between 250 cm^−1^ and 4000 cm^−1^ wave numbers [29,31–33]. Figure 3 shows the FTIR spectra of initiate precipitate. In this figure, the absorption peaks at 3365.17 cm^−1^ and 1650.34 cm^−1^ present the functional unit of O–H and C=O bonds relate to moisture in sample and bond in PVP respectively. The absorption peaks at 687.62, 953.71 and 1345.78 attribute to borate network according to Table 3.

The vibration modes of the borate network are mainly active in three IR spectra region: 850–1100 cm^−1^ associated with the B–O stretching of tetrahydral BO^4−^ units, and 600–800 cm^−1^ associated with bending vibrations of various borate segments and a band at around 700 cm^−1^, which corresponds to bond bending of B–O–B bridges in the boron-oxygen network [29,31–34].

**Figure 4:**
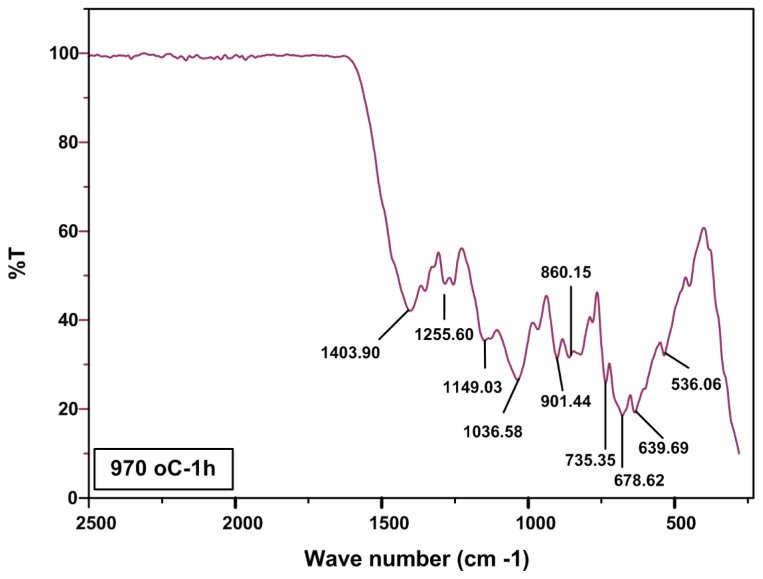
IR spectra of calcium borate nanoparticles for annealed samples at 970 °C.

The IR spectra of annealed sample at 970 °C are shown in Figure 4. It is clear that the all observed peaks in this figure relates to borate network and the peaks of O–H and C=O disappears due to evaporation of moisture and decomposition of PVP.

### 2.3. TEM Results

**Figure 5:**
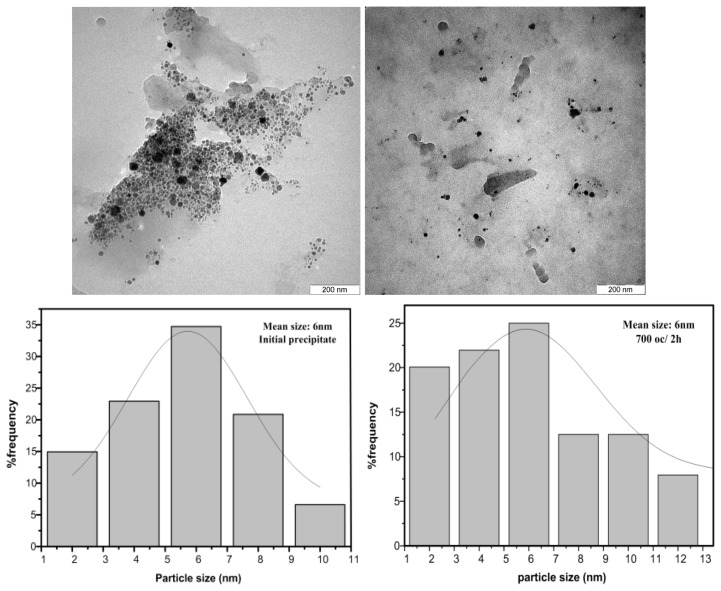

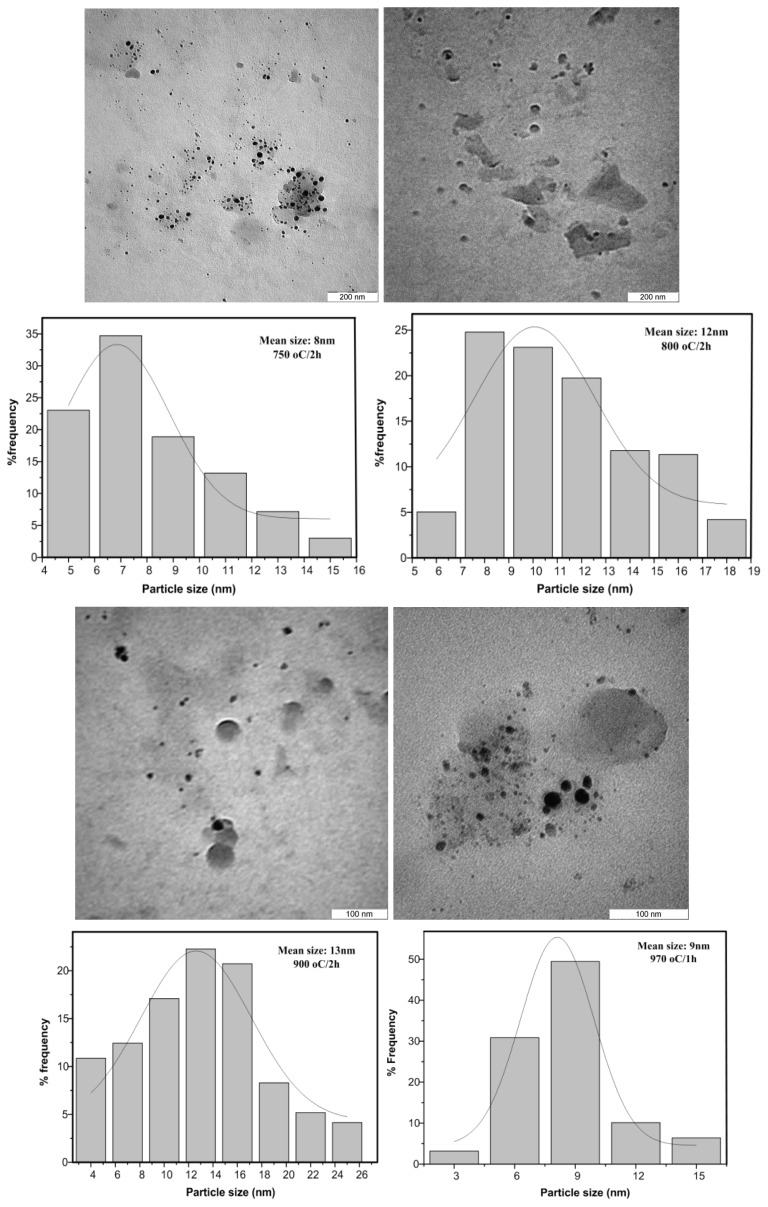
Transmission electron microscopy (TEM) image and size distributions of calcium borate nanoparticles anealed at different temperatures for fixed time of 2 h.

Transmission electron microscope (TEM) was carried out on the powder samples to monitor their particle size and the morphology. The average sizes and size distribution of nanoparticles were evaluated from TEM images using UTHSCA Image Tool considering at least 200 nanoparticles for each sample. The results represent that the particles have spherical shapes with relatively homogenous distribution (Figure 5). The figure shows that both initial precipitate and annealed samples at 700 °C for 2 h have the smallest average size of 6 nm, but the annealed samples has broader size distribution which is due to further growth of nanoparticles during heating process. With increasing the temperature, the particle sizes increment moderately so that, the average size reaches to 14 nm for annealed samples at 970 °C for 2 h.

Another factor that affected particle size and size distribution is annealing time. As can be seen from Table 1, the increasing of time has significant effect on enlargement of particle size, although it had less effect on conversion of crystal structure. The maximum average particle size of 15 nm is obtained after annealing at 800 °C for 5 h. This suggests that the several neighboring particles fuse together to increase the particle size by the melting of their surfaces [35].

### 2.4. TGA Analysis (Thermal Stability)

To assess the thermal behavior of Calcium borate, the samples were subjected to thermogravimetric analysis (TGA). TGA measures the rate and amount of change in the weight of a material as a function of temperature in the inert atmosphere. The measurements were carried out at room temperature up to 1100 °C.

**Figure 6:**
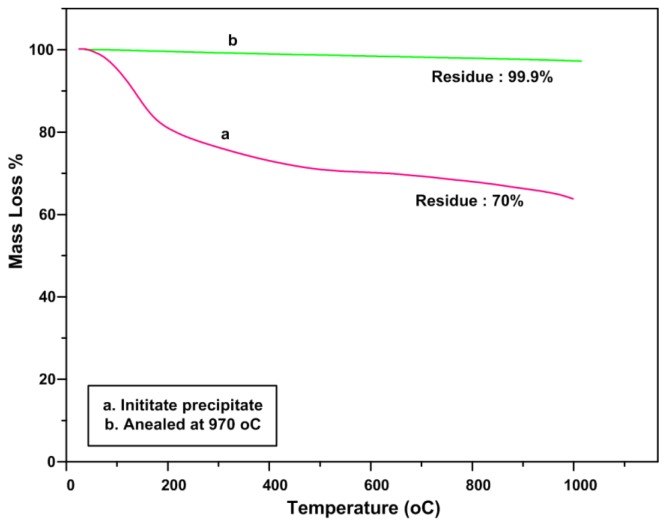
Thermal stability of calcium borate at initiate precipitate and annealing at 970 °C.

The result shows the mass loss of 30% for initiate precipitate at extended temperatures between 126 °C and 500 °C. This huge mass loss is most probably due to trapped moisture in the sample and decomposition of PVP which is in agreement with FTIR results (Figure 6a). The calcium borate annealed at 970 °C retains the original symmetry when heated up to 1100 °C (Figure 6b). It shows only one negligible mass loss at around 87 °C which is probably due to trapped moisture in the sample.

A high concentration of surface atoms and defects at nanophosphors materials can be regarded as one of the fundamental characteristics which have an effective role in luminescence properties. The created surface charge-carrier trapping centers in nanomaterials has different energy depth from analogous centers in bulk materials [7] and the carrier recombination rate increases due to the increase of the overlap between the electron and the hole wave functions [36]. The borate compounds, which have an effective atomic number very close to that of human tissue (*Z*_eff_ = 7.42), are promising materials to develop for use in radiation monitoring in medical applications [13].

## 3. Experimental Details

The starting materials for the synthesis of calcium borate were Calcium chloride (CaCl_2_) and borax (Na_2_B_4_O_7_) as the precursors and polyvinyl pyrrolidone (PVP) as the capping agent which were purchased from sigma Aldrich Co. All the chemicals were of analytical re-agent grade and used without further purification.

In a typical procedure, 1 g of PVP (MW: 10000) was dissolved in 100 mL de-ionized water. Subsequently, 0.2 mol% of calcium chloride was added to PVP solution so as to obtain homogeneous solution. Moreover, the borax solution was prepared separately by adding 0.2 mol% of borax into glass beaker of 100 mL de-ionized water. Both solutions heated to 48 °C and stirred for 1 h. The calcium solution was then mixed with borax solution drop wise very slowly. A very fine white precipitate appears after a short time according to the following chemical reaction [37]:

$${\text{Na}}_{2}{\text{B}}_{4}{\text{O}}_{7}\cdot 10{\text{H}}_{2}0+{\text{CaCl}}_{2}\to \text{CaO}.2{\text{B}}_{2}{\text{O}}_{3}\cdot 10{\text{H}}_{2}\text{O}↓+2\text{NaCl}$$

The solution was stirred for extra 1 h in order to complete the reaction and get better homogenous particles. Afterwards, the precipitates was centrifuged and washed several times with distill water. The white precipitated Calcium borate nanoparticles were dried at 80 °C for 24 h. The precipitate was centrifuged and washed several times with distill water and dried at 353 K for 24 h. Further annealed at different temperatures and time were carried out in a resistive furnace in air for formation of nanocrystals. The products were characterized by X-ray powder diffraction (XRD) at a scanning rate of 5°/min in the 2*θ* range 4°–70° using a Shimadziu X-ray diffractometer (XRD 6000) with Cu Kα radiation (λ = 0.1542 nm). The vibrational modes of functional groups of the produced phases were determined by using Fourier transform infrared (FTIR) spectrometer (Pekin Elmer model 1650). The FTIR spectra were recorded in 280 cm^−1^–4000 cm^−1^ region. The particle size and size distribution were determined from the transmission electron microscopy (TEM) micrographs (JOEL 2010F UHR) operating at 200 kV. The average size and size distribution of nanoparticles were evaluated in TEM images using UTHSCA Image Tool considering at least 200 nanoparticles for each sample.

## 4. Conclusion

The nanocrystalline calcium borate has been prepared successfully by co-precipitation method followed by thermal treatment method. The transformation to crystalline phase originated at annealing temperature of 700 °C for 5 h annealing time with the formation of metaborate and the formation of tetraborate phase was observed at 970 °C. The average particle sizes are in the range of 6 nm to 15 nm which obtained in different annealing time and temperature, as confirmed by TEM analysis. The thermal stability of nanocrystals improved when they annealed at a higher temperature.

## Figures and Tables

**Table 1:** The main diffraction peaks related to dominate phases at different annealing temperatures at 2 h annealing time.

Temperature	Main Diffraction Peaks	h	k	l	Dominate Phase
970 °C	24.42	0	1	2	CaB_4_O_7_
	26.092	−3	1	1	
	26.47	−2	0	1	
	29.75	2	1	0	
	46.89	4	3	2	
900 °C	26.40	1	1	1	CaB_2_O_4_
	29.75	2	1	0	
	34.41	1	3	1	
	42.23	0	0	2	
	46.85	1	5	1	
800 °C	19.85	1	0	2	CaB_2_O_4_
	29.81	2	1	0	
750 °C	19.85	1	0	2	CaB_2_O_4_
	29.81	2	1	0	

**Table 2:** Variation of annealing temperatures, annealing times, particle sizes, and crystallinity of calcium borate nanoparticles.

Sample	Annealing Temperature (°C)	Annealing Time (h)	TEM Size (nm)	Dominate Phase
1	0	0	6	Amorphous
2	700	2	6	Amorphous
3	700	3	9	Amorphous
4	700	5	13	CaB_2_O_4_
5	750	2	8	CaB_2_O_4_
6	750	3	11	CaB_2_O_4_
7	750	5	15	CaB_2_O_4_
8	800	2	12	CaB_2_O_4_
9	800	3	13	CaB_2_O_4_
10	800	5	15	CaB_2_O_4_
11	900	1	13	CaB_2_O_4_
12	900	2	14	CaB_2_O_4_
14	970	1	9	CaB_4_O_7_
15	970	2	14	CaB_4_O_7_

**Table 3:** Frequencies and their assignments for IR spectra of CaB_4_O_7_.

Frequencies (cm^−1^)	Assignment	Reference
~685	B–O–B bending vibration	[29]
~700	B–O–B bending vibration in borate ring	[29,31,33]
850–1100	B–O stretching of tetrahydral BO_4_^−^	[29,31,33]
600–800	bending vibrations of various borate	[31,33]
~907	B–O streching vibration of BO_4_ units in tri, tetra and pantaborate groups	[29,32]
1400	B–O stretching trigonal BO_3_ units	[29,31,33,34]
